# Differential gene expression in response to eCry3.1Ab ingestion in an unselected and eCry3.1Ab-selected western corn rootworm (*Diabrotica virgifera virgifera* LeConte) population

**DOI:** 10.1038/s41598-019-41067-7

**Published:** 2019-03-20

**Authors:** Zixiao Zhao, Lisa N. Meihls, Bruce E. Hibbard, Tieming Ji, Christine G. Elsik, Kent S. Shelby

**Affiliations:** 1 Division of Plant Sciences, University of Missouri, Columbia, MO USA; 2 USDA-ARS, Columbia, MO, Columbia, MO USA; 3 Department of Statistics, University of Missouri, Columbia, MO USA; 4 Division of Animal Sciences, University of Missouri, Columbia, MO USA; 5 MU Informatics Institute, University of Missouri, Columbia, MO USA

## Abstract

*Diabrotica virgifera virgifera* LeConte, the western corn rootworm (WCR) is one of the most destructive pests in the U.S. Corn Belt. Transgenic maize lines expressing various Cry toxins from *Bacillus thuringiensis* have been adopted as a management strategy. However, resistance to many Bt toxins has occurred. To investigate the mechanisms of Bt resistance we carried out RNA-seq using Illumina sequencing technology on resistant, eCry3.1Ab-selected and susceptible, unselected, whole WCR neonates which fed on seedling maize with and without eCry3.1Ab for 12 and 24 hours. In a parallel experiment RNA-seq experiments were conducted when only the midgut of neonate WCR was evaluated from the same treatments. After *de novo* transcriptome assembly we identified differentially expressed genes (DEGs). Results from the assemblies and annotation indicate that WCR neonates from the eCry3.1Ab-selected resistant colony expressed a small number of up and down-regulated genes following Bt intoxication. In contrast, unselected susceptible WCR neonates expressed a large number of up and down-regulated transcripts in response to intoxication. Annotation and pathway analysis of DEGs between susceptible and resistant whole WCR and their midgut tissue revealed genes associated with cell membrane, immune response, detoxification, and potential Bt receptors which are likely related to eCry3.1Ab resistance. This research provides a framework to study the toxicology of Bt toxins and mechanism of resistance in WCR, an economically important coleopteran pest species.

## Introduction

The Bt (*Bacillus thuringiensis*) Cry3 δ-endotoxin superfamily is known for its specificity to coleopteran species^1,2^. Transgenic maize hybrids expressing Cry3Bb1, mCry3A, eCry3.1Ab as well as Cry34/35Ab1 have been introduced to the market to manage western corn rootworm (WCR, *Diabrotica virgifera virgifera* LeConte). As a highly adaptive species, WCR has developed resistance to broadcast soil insecticides^3^, aerial spray insecticides for reducing adult number^4,5^, crop rotation^6^, and Cry3 Bt toxins. Laboratory selection experiments have developed WCR colonies resistant to Cry3Bb1, mCry3A, Cry34/35Ab1 and eCry3.1Ab by continuously rearing WCR on transgenic maize lines^7–10^. Resistance to Cry3 Bt toxins has become a practical issue since field resistant WCR populations have been reported in many locations^11–14^. Although eCry3.1Ab is the most recent Bt toxin^15^ to the market without reported control failure in the field, laboratory selection^9^ and cross-resistance experiments^13^ indicate that resistance to eCry3.1Ab is likely in the field due to the resistance to other Cry3 proteins^16^.

Three Cry toxins (Cry3Bb1, mCry3A and eCry3.1Ab) for WCR control are derived from the Cry3 superfamily. In lepidopteran systems, the consumption of Bt protoxin by larvae is followed by activation *via* cleavage by midgut proteases, binding to brush border receptors, toxin insertion into the epithelial cell membrane, pore formation by oligomerization, and finally, destruction of midgut epithelial cells^17,18^ followed by fatal sepsis^19^. Anything interfering with these processes (*e.g*. decreased activation, reduced receptor affinity, increased toxin degradation, or increased repair of midgut epithelial cells) could cause resistance^20^. However, the detailed interactions between Cry3 toxins and WCR is less known. Similar to Lepidoptera, reduced binding of mCry3A to the midgut epithelial cell membrane has been observed in resistant WCR colonies^21^. However, no Cry3 receptor has been validated in Coleopterans. Although potential receptors including cadherin-like protein^22^ and ATP-binding cassette (ABC) transporters^23^ are present in WCR, their roles in Bt resistance are questionable due to the lack of direct evidence of protein interactions.

Toxin dose is a very important component of what has been coined the ‘high-dose/refuge strategy’ to delay resistance^14^. In this theory the ‘high-dose’ refers to Bt toxins that cause very high mortality to pest populations. The initial frequency of alleles conferring resistance is low, fitness costs of resistance are present, mating between resistant and susceptible insects is random, and resistance to the Bt toxin is recessive^24,25^. For lepidopteran pests, for instance *Heliothis virescens* (Fabricius), the Cry1Ac is considered as a high-dose toxin, which is able to kill the insects with susceptible alleles. Only individuals with homozygous resistant alleles will survive, yielding a population with an extremely high resistance ratio to Bt toxin. The resistant genes could be identified by QTL mapping^26^ from a homozygous resistant population. In WCR, the situation is less optimal. All four WCR-targeting Bt toxins are considered as low to moderate dose^27–30^, where individuals heterozygous for resistance alleles may survive. Second, previous studies showed resistance to Bt in WCR may be caused by multiple genes^23^. The resistant genes selected in laboratory-selected resistant populations may not be sufficient to estimate the resistant alleles in field resistant populations due to founder effects. Third, the lack of a well-assembled, high-quality WCR genome sequence hampers the application of advanced genetic and molecular approaches to analyze resistant-related variation at the genomic level. RNA-seq followed by *de novo* assembly is an alternative for analyzing gene expression without a reference genome. In WCR, RNA-seq has been successfully used to study callus digestion^31^, interaction with corn defense chemicals^32^, adaptation to pesticides^33^, resistance to crop rotation^34^ and response to Cry34/35Ab1^35^, and Cry3Bbb1^36^.

To investigate the gene response to eCry3.1Ab intoxication and mechanisms of resistance, we sequenced the transcriptome of eCry3.1Ab-selected resistant and unselected, susceptible WCR feeding with maize root with and without eCry3.1Ab for 12 and 24 hours. Moreover, to further track down the tissue interactions with Bt toxin we sequenced the transcriptome of midgut from recovered neonates with the same treatment. The expression patterns of different populations under these conditions reveal how WCR respond to eCry3.1Ab and provide clues of resistance mechanisms.

## Results and Discussion

### Transcriptome assembly and annotation of WCR transcriptome

**Figure 1:**
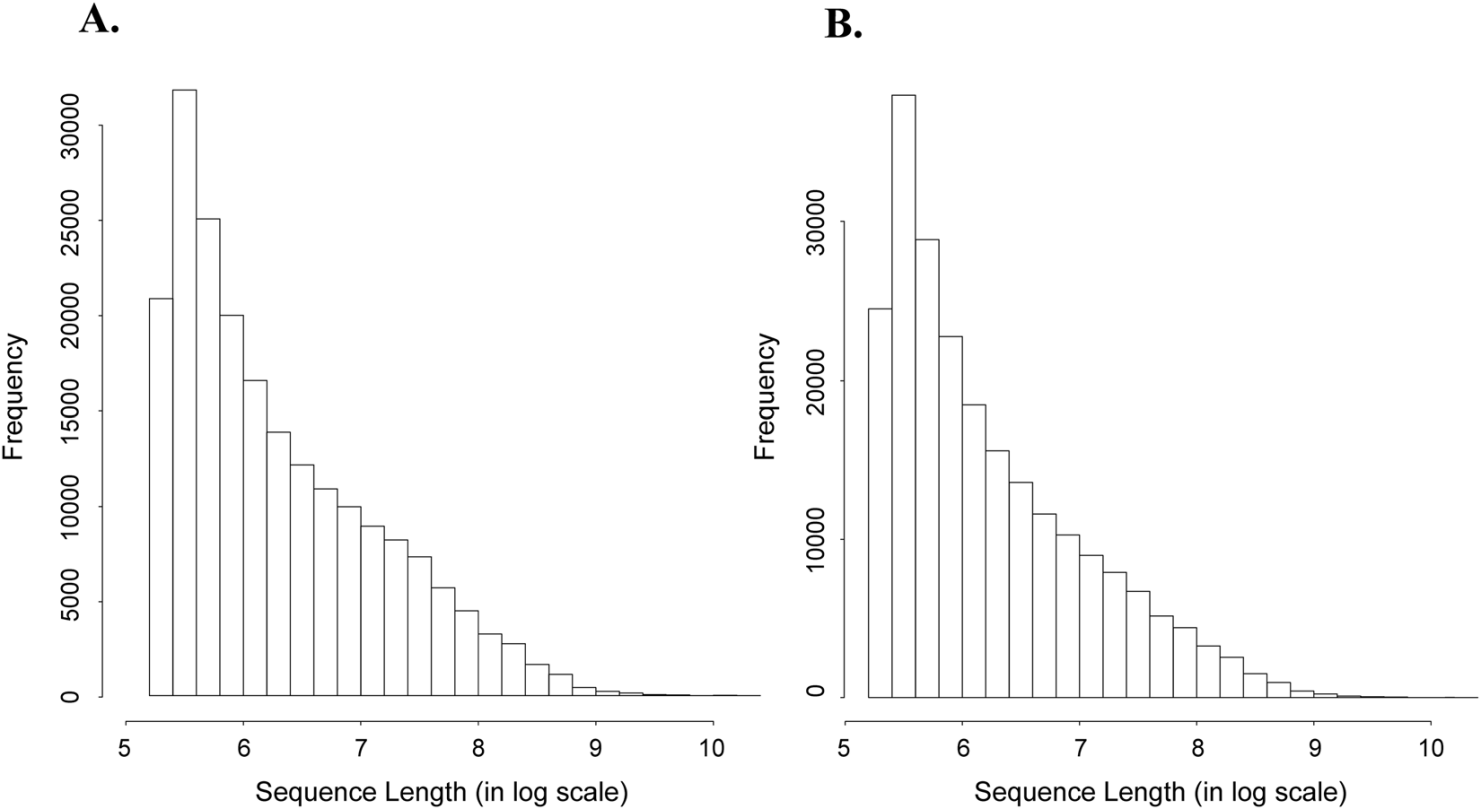
Contig length distribution of WCR (**A)** larval and (**B**) midgut transcriptome assemblies. The length of each contig has been converted to log scale.

The transcriptome of whole larvae and midgut were separately *de novo* assembled. Reads from either whole larvae or midgut were individually pooled to increase the assembly coverage. Low quality and mitochondrial sequences were removed prior to assembly. Transcriptome assembly resulted in a whole larval transcriptome with 204,842 contigs from 57 Gb of reads and midgut transcriptome with 226,115 contigs from 137 Gb of reads. The two transcriptomes had comparable average sequence length (measured as N50, a weighted median statistic that more than half of the nucleotides of a transcriptome belong to the contigs of this size N50 or longer), GC content and length distribution (Table 1, Fig. 1). After removing duplicate contigs with more than 95% sequence similarity, we obtained 187,570 and 209,167 contigs respectively from larval and midgut transcriptome (Table 1). Herein, we refer to the two reduced redundancy sets of contigs as “unigene” sets. BUSCO^37^ was applied to evaluate the completeness of each unigene set. All of the transcriptome and unigene sets cover over 95% of insecta single-copy orthologs. Both unigene sets maintain the coverage and integrity while duplication is reduced, indicating the acceptable qualities for functional analysis (Table 2).

**Table 1:** Summary statistics of WCR whole larvae and midgut transcriptome assemblies and their unigene sets.

	Whole Larval Transcriptome		Midgut Transcriptome	
**Input Reads**				
Raw reads pair	313,684,316		750,660,524	
Filtered reads pair	284,268,606		727,149,108	
Filtered total bases	57,171,701,994		137,820,263,438	
**Transcriptome Statistics**				
	**Trinity Assembly**	**Unigene Set**	**Trinity Assembly**	**Unigene Set**
Total assembled bases	173,452,950	130,813,521	176,268,219	136,641,964
Number of contigs	204,842	187,570	226,115	209,167
Average contig length	847	697.4117	780	653.2673
Min contig length	201	201	201	201
Max contig length	31,383	31,119	28,475	27,353
Number of contigs > 1 kb	48,389	34,466	46,762	33,637
Number of contigs > 5 kb	2,584	1,014	2,346	946
Number of contigs > 10 kb	216	59	191	56
N50	1,523	1,096	1,351	977
GC content (%)	35.91	35.45	36.54	36.36

**Table 2:** Summary of BUSCO analysis of WCR whole larval and midgut transcriptome assemblies and their unigene sets.

	Whole Larvae		Midgut	
	Trinity Assembly	Unigene Set	Trinity Assembly	Unigene Set
Complete (%)	95.7	95.5	96.6	96.6
Duplicated (%)	27.6	21.7	31.2	23.7
Fragment (%)	3.3	3.5	1.7	1.7
Missing (%)	1.0	1.0	1.7	1.7

**Figure 2:**
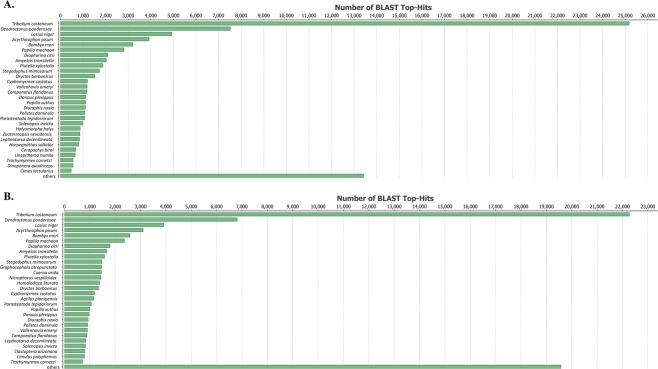
The species distribution of BLASTX top hits of WCR (**A**) larval and (**B**) midgut transcriptome assemblies.

Transcriptomes and their unigene sets were aligned to the NCBI non-redundant protein database (NR) using BLASTX. Only 42% of larval and 38.2% of midgut unigenes had significant BLASTX results. The species distribution of BLASTX top hits indicated that a predominant number of unigenes could be annotated by the Coleopteran model species *Tribolium castaneum* (Herbst) (Table 3, Fig. 2). We also identified 372 and 400 unigenes from larval and midgut transcriptome, respectively, that were directly aligned to 13 known genes from *Diabrotica* species (Tables 3 and 4).

**Table 3:** Summary of BLASTX of WCR whole larval and midgut transcriptome assemblies and their unigene sets.

	Whole Larvae		Midgut	
	Trinity Assembly	Unigene set	Trinity Assembly	Unigene set
Total Contigs for BLASTX	204,842	187,570	226,115	209,167
Contigs with BLASTX Hits	86,123	73,699	86,411	75,388
Contigs with Coleopteran Hits				
*Diabrotica* spp.	292	372	315	400
*Tribolium castaneum*	25,617	34,936	22,262	33,924

**Table 4:** Existing *Diabrotica* spp. genes identified in WCR whole larval and midgut transcriptomes.

Type	Accession #	Description
Midgut	ANW44175.1	Spectrin alpha chain-like protein, partial
Midgut	ANX99824.1	Ribosomal protein S10
Midgut	ANX99822.1	Proteasome subunit beta type-1-like protein
Midgut	ANX99823.1	Proteasome subunit alpha type-3-like protein
Midgut	ANX99821.1	Smooth septate junction protein 2
Midgut	AHJ09935.1	Glycoside hydrolase family 28
Midgut	AAF87760.1	Cytochrome oxidase subunit I, partial (mitochondrion)
Midgut	AGF33977.1	Cytochrome oxidase subunit I, partial (mitochondrion)
Midgut	ABU50691.1	Cadherin-like protein, partial
Larvae	YP_008854784.1	NADH dehydrogenase subunit 1 (mitochondrion)
Larvae	AHA51728.1	NADH dehydrogenase subunit 5 (mitochondrion)
Larvae	AHA51723.1	Cytochrome c oxidase subunit 2 (mitochondrion)
Larvae	AHA51722.1	Cytochrome c oxidase subunit 1 (mitochondrion)

### Differential expression analysis of WCR transcriptome

**Figure 3:**
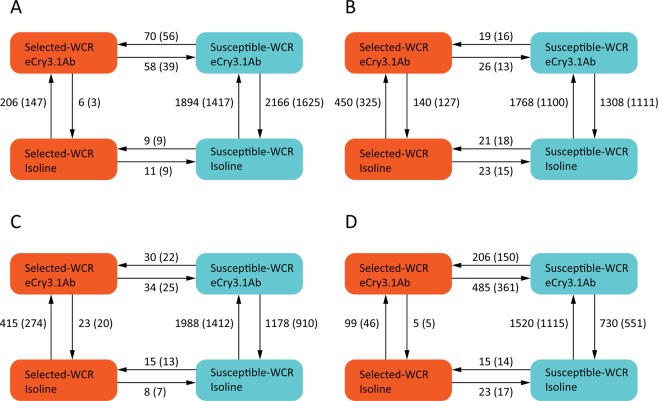
Transcriptional response of neonate WCR allowed feed 12 or 24 hrs on eCry3.1Ab or non-Bt isoline maize seedlings compared to resistant neonates feeding on isoline seedlings. R: eCry3.1Ab resistant WCR; S: susceptible WCR; Bt: eCry3.1Ab transgenic seedlings; Isoline: non-Bt seedlings; Differential expression pattern: (**A**) larval response at 12 hours. (**B**) Larval response at 24 hours. (**C**) Midgut response at 12 hours. (**D**) Midgut response at 24 hours (adjust p-value < 0.05, FDR test).

**Figure 4:**
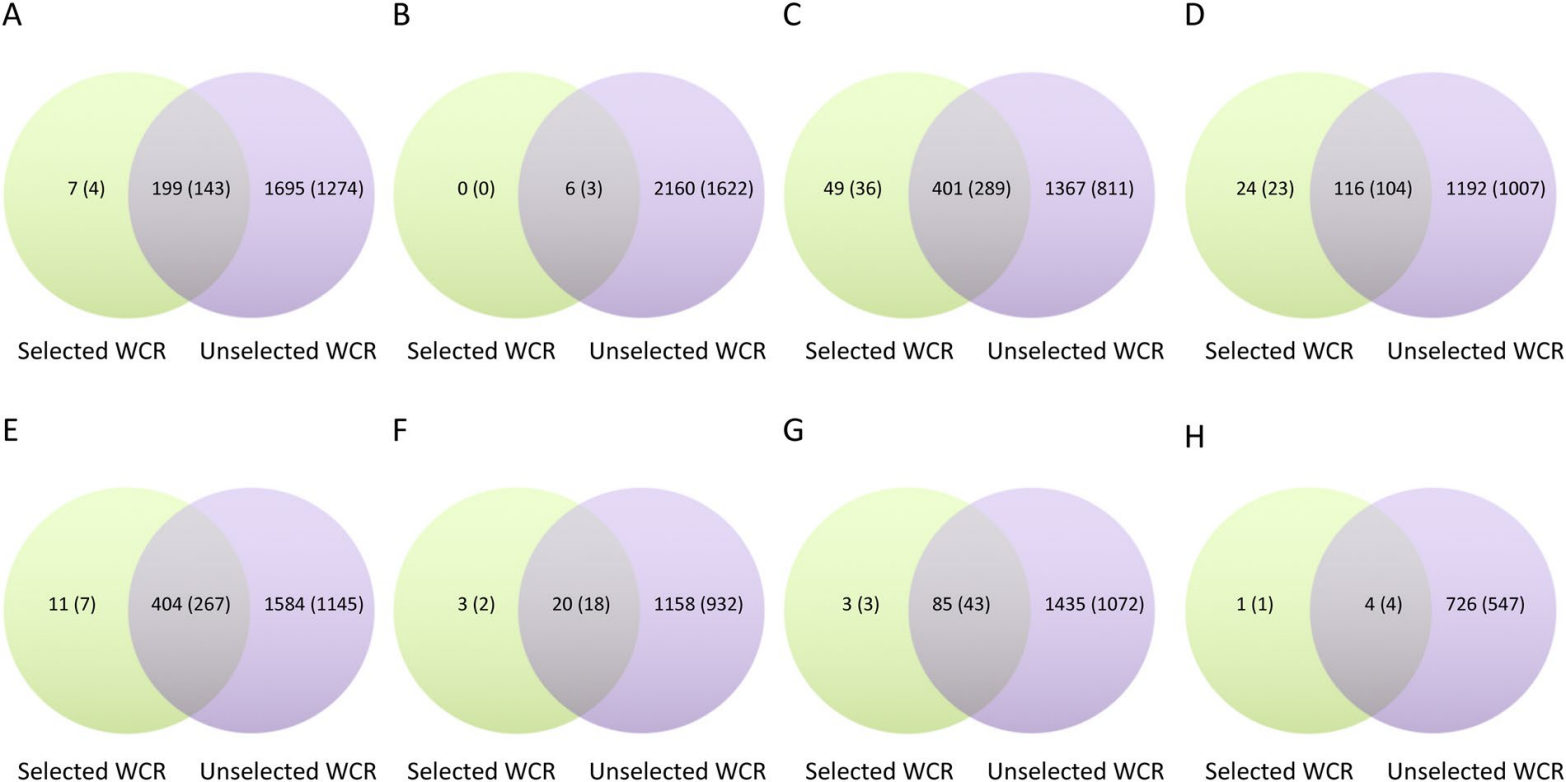
Differential expression pattern of resistant and susceptible WCR in whole larvae, or dissected midguts, when feeding on Cry3.1Ab transgenic maize seedlings vs. feeding on non-Bt isoline seedlings, for 12 or 24 hrs. In whole larvae (**A**,**B**,**C** and **D**) at 12 hrs feeding up-regulated (**A**), and down-regulated (**B**) contigs, showing overlapping expression. In whole larvae at 24 hrs feeding up-regulated (**C**), and down-regulated (**D**). In dissected midgut tissues (**E**,**F**,**G** and **H**) contigs up-regulated at 12 hr feeding (**E**), and down-regulated (**F)**. At 24 hrs feeding contigs up-regulated (**G**) and down-regulated (**H**). Numbers in shown in parentheses are contigs with BLASTX annotation.

To identify the genes involved in eCry3.1Ab response, we analyzed the differentially expressed unigenes from whole larval and midgut transcriptomes between eCry3.1Ab-feeding and non-Bt isoline feeding WCR at both 12- and 24-hour time points. To understand the expression differences between eCry3.1Ab-selected and susceptible, control WCR from the same original population, we also compared the expression differences in whole larvae and midgut between two populations when being fed with the same kind of maize root. After the alignment and filtering out unigenes with extremely low expression levels, only 31,875 of larval and 22,954 of midgut transcriptome unigenes were proceeded to edgeR analysis^38^. The patterns of differentially expressed genes are shown in Fig. 3. Regardless of exposure time or tissue type, susceptible WCR differentially expressed a much larger number of genes in response to eCry3.1Ab intoxication. In contrast, eCry3.1-selected WCR had dramatically fewer differentially expressed genes (DEGs). The selected and unselected WCR shares many DEGs in both whole larvae and midgut transcriptome, while some unique DEGs were colony specific, especially in unselected colony (Fig. 4).

Albeit smaller in size, we identified DEGs between eCry3.1Ab-selected and unselected WCR, especially when both groups of neonates were fed on eCry3.1Ab maize root. The function of those DEGs and the pathways in which they were involved may reveal the physiological differences and the mechanism of eCry3.1Ab resistance in the selected population.

### GO annotation and pathway analysis on eCry3.1Ab feeding WCR midgut

To further investigate the molecular and physiological adaptation to intoxication on eCry3.1Ab-selected WCR we applied gene ontology (GO) analysis to differentially expressed genes using Blast2GO. We compared the top 20 level-2 GO terms called from the unigene set from midgut DEGs between selected and unselected WCR feeding on eCry3.1Ab maize for 24 h. The GO terms were ranked based on number of unigenes of each GO term. The terms “metabolic process”, “catalytic activity” and “membrane” accounted for the majority of each of the three ontologies [Biological Process (BP), Molecular Function (MF) and Cellular Component (CC), respectively], suggesting the primary location and functions of eCry3.1Ab resistance. Other than that, the BP term “cellular process” and MF term “binding” also implied the binding and processing of toxin may have a role in eCry3.1Ab sensitivity.

We compared the distribution of level-2 GO terms between the DEG unigene set described above and midgut transcriptome unigene set (Table 5). The BP terms “Multiple-organism process”, “immune system process”, “detoxification” and “cell killing” and the CC terms “extracellular region” and “extracellular part” were among the most over represented GOs in DEG unigene sets, while the BP term “reproduction” and MF term “structural molecule activity” were the most under represented. However gene set enrichment analysis (GSEA) showed that no specific GO term was significantly enriched in the DEG unigene set.

**Table 5:** The top 20 level-2 GO terms of DEG unigene set and midgut transcriptome unigene sets.

	DEG Unigene set		Midgut Unigene Set	
***Biological Process***				
**GO Terms**	**Percentage**	**Rank**	**Percentage**	**Rank**
metabolic process	29.52%	1	8.56%	1
single-organism process	15.05%	2	3.78%	3
cellular process	13.60%	3	8.09%	2
localization	7.09%	4	1.58%	6
biological regulation	2.75%	5	1.98%	4
response to stimulus	2.17%	6	1.32%	7
regulation of biological process	2.03%	7	1.79%	5
multicellular organismal process	1.45%	8	0.54%	10
developmental process	1.16%	9	0.47%	11
cellular component organization or biogenesis	0.87%	10	1.01%	8
signaling	0.87%	10	0.92%	9
negative regulation of biological process	0.72%	12	0.21%	13
positive regulation of biological process	0.58%	13	0.21%	12
immune system process	0.58%	13	0.11%	18
multi-organism process	0.43%	15	0.14%	16
detoxification	0.29%	16	0.03%	22
cell killing	0.29%	16	0.01%	24
reproductive process	0.14%	18	0.14%	15
reproduction	0.14%	19	0.14%	14
locomotion	0.14%	19	0.09%	19
***Molecular Function***				
**GO Terms**	**Percentage**	**Rank**	**Percentage**	**Rank**
catalytic activity	32.85%	1	8.52%	2
Binding	20.98%	2	8.79%	1
transporter activity	3.62%	3	0.83%	3
molecular transducer activity	0.72%	4	0.27%	6
molecular function regulator	0.43%	5	0.23%	7
structural molecule activity	0.43%	5	0.82%	4
electron carrier activity	0.43%	5	0.08%	9
signal transducer activity	0.29%	8	0.30%	5
antioxidant activity	0.29%	8	0.05%	10
***Cell Compartment***				
**GO Terms**	**Percentage**	**Rank**	**Percentage**	**Rank**
membrane	9.12%	1	3.64%	3
cell	5.93%	2	4.14%	1
cell part	5.79%	3	4.09%	2
extracellular region	5.35%	4	0.25%	9
membrane part	5.07%	5	2.43%	5
organelle	4.05%	6	2.69%	4
extracellular region part	2.46%	7	0.09%	11
organelle part	1.88%	8	1.43%	7
macromolecular complex	0.87%	9	1.74%	6
supramolecular complex	0.43%	10	0.10%	10
membrane-enclosed lumen	0.43%	10	0.37%	8

The DEGs are from comparison of midgut gene expression between select and unselected-WCR when both were given eCry3.1Ab-expressing roots for 24 hours. The GO terms are ranked by number of sequences of each GO, as well as the percentage of sequences among each unigene set.

We further predicted enzyme codes (EC) from GO terms for unigenes and used the EC to map differentially expressed unigenes to pathways from the Kyoto Encyclopedia of Genes and Genomes (KEGG) database^39^. The differentially expressed midgut unigenes were involved in 49 pathways, including purine metabolism (KEGG ID: 00230), glutathione metabolism (KEGG ID: 00480), fatty acid synthesis (KEGG ID: 00061), glycerophospholipid metabolism (KEGG ID: 00564) and drug metabolism (KEGG ID: 00983). The fatty acid synthesis pathway was also identified in whole larval differentially expressed unigenes. These results suggest that differences in eCry3.1Ab tolerances might arise from the alternation of genes related to detoxification, membrane functions and metabolism.

### Expression of potential and novel eCry3.1Ab resistant genes

Research has revealed that cadherins^26^, ABC transporters^40^, and aminopeptidase N (APN)^41^ are Bt receptors in lepidopteran insects. Both cadherins and ABC transporters have been found in WCR^22,23^. Upon BLASTX, we found 102 unigenes with cadherin function, 209 unigenes with ABC transporter function, and 50 unigenes with APN function from the midgut unigene set. In whole larval unigene set, there were 78 unigenes with cadherin function, 191 unigenes with ABC transporter function, and 62 unigenes with APN function.

**Figure 5:**
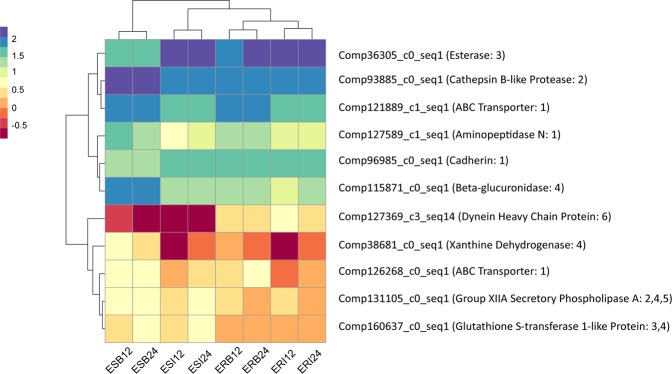
Expression levels of 11 potential eCry3.1Ab resistance related genes in 8 WCR midgut treatment group (see Table 6). Expression level s are quantified by count per million read (cpm). The candidate genes are categorized into: 1: potential Bt receptors; 2: digestive proteases; 3: detoxification enzymes; 4: enzymes involve in drug metabolism pathway; 5: enzymes involve in membrane related pathways; 6: other candidates.

The midgut gene expression profile showed that cadherin was not significantly differentially expressed, while some ABC transporters (multidrug resistance-associated protein) and APNs were differentially expressed following Cry3.1Ab ingestion, especially in susceptible WCR (Table 6, Fig. 5). The expression level of one APN midgut unigene (comp127589_c1_seq. 1) was significantly higher in susceptible WCR midgut, when both selected and susceptible-WCR were given eCry3.1Ab for 24 hours. Two ABC transporter unigenes (comp121889_c0_seq. 1, comp126268_c0_seq. 1) showed the same pattern, but the increased expression in susceptible WCR was not significant. The protein structure of eCry3.1Ab toxin contains partial Cry1Ab sequence at its C-terminus^15,42^. In multiple lepidopteran species APNs serve as binding receptors of Cry1A toxins and mutations or reduced expression of APN result in resistance to Bt toxins^41^. Our results suggest that APN is a potential eCry3.1Ab target and the reduced expression level under intoxication might contribute to resistance in WCR.

**Table 6:** Description of treatment groups for WCR larvae shown in Fig. 5.

Treatment Code	WCR colony	Maize Seedling Type	Feeding period (hr)
ERB12	Selected resistant	eCry3.1Ab	12
ERB24	Selected resistant	eCry3.1Ab	24
ERI12	Selected resistant	Non-Bt Isoline	12
ERI24	Selected resistant	Non-Bt Isoline	24
ESB12	Unselected susceptible	eCry3.1Ab	12
ESB24	Unselected susceptible	eCry3.1Ab	24
ESI12	Unselected susceptible	Non-Bt Isoline	12
ESI24	Unselected susceptible	Non-Bt Isoline	24

Proteases have been associated with Bt resistance either by increasing digestion of toxins^43,44^, or by decreasing the proteolytic activation of Bt pro-toxins^45^. We identified 112 unigenes with metalloprotease functions and 199 unigenes with cathepsin functions in the larval unigene set. In the midgut unigene set, there were 131 unigenes with metalloproteases functions and 234 unigenes with cathepsin functions. Expression levels of some proteases were regulated by Bt intoxication either in selected or susceptible WCR, while none of those digestive proteases was up regulated in selected WCR midgut compared to the susceptible one when both insects were fed with eCry3.1Ab maize.

Considering the expression patterns, GO annotation, and pathway analysis, we infer that at least two novel genes are likely involved in the resistance to eCry3.1Ab. Esterase has been reported involved in Cry1Ac resistance in *Helicoverpa armigera*^46^. In WCR, we observed a novel esterase (comp36305_c0_seq. 1) expressed in the midgut of selected colony was higher than unselected one after 24 hours of intoxication. The second gene is a dynein heavy chain-like protein (comp127369_c3_seq. 14). It was constitutively up regulated in selected WCR regardless of diet and time. Since dynein is a cytoskeletal motor protein involved in intracellular transportation and the movement of chromosomes, we propose that selected-WCR many have a stronger activity in either endocytosis or cell mitosis to remove the attached eCry3.1Ab molecules^47^, or to repair damaged epithelial cells^48^.

## Conclusions

After a comprehensive analysis of an RNA-seq experiment with eCry3.1Ab-selected and susceptible, control WCR, the transcriptome, unigene sets and reads provided numerous resources for studying the interaction between Cry3 and this coleopteran species. This study is the first step approaching the delineation of Bt resistance mechanisms in WCR. We propose more than one potential mechanism of resistance to eCry3.1Ab – a dual action Bt toxin. With the recently published WCR genome sequence, future research will detect the genomic-wide genetic variations associated with Bt resistance. Studies to explore how Bt toxins affect gene alternative splicing and whether the alternatively sliced genes are related to Bt resistance in WCR. We are also developing cellular and molecular methods *i.e*. cultured cell expression, RNAi gene silencing, individual genotyping to further study the detailed mechanism of resistance to eCry3.1Ab as well as other Bt toxins. Continuous discoveries in this field will lead to improving strategies for insect resistant management and the developing of novel entomotoxins.

## Material and Methods

### Insects and bioassay

The eCry3.1Ab-selected resistant WCR colony was initially selected and reared on non-elite non-commercial eCry3.1Ab-expressing transgenic maize (event 5130) under laboratory conditions^9^. Both the selected and susceptible control colonies were developed from a single population and had been maintained on eCry3.1Ab-expressing transgenic maize (material ID 12MG00345) and its near-isoline (material ID 12MG001181), respectively, for more than 30 generations. For the current experimental design (Supplementary Table 1, column 1), both eCry3.1Ab-expressing and isoline maize seeds were surface sterilized and germinated in Petri dishes with moistened filter paper at 23 °C for 4–6 days without illumination. Approximately 30 neonates hatched within 24 hours were transferred to a Petri dish containing 3–4 seedlings of each line. After 12 or 24 hours feeding, the living first-instar larvae were recovered. In a separate identical experiment (Supplementary Table 1, column 2), the midgut was dissected from 30–40 recovered larvae of each Petri dish. Both whole larvae and midgut were flash frozen in liquid nitrogen and stored at −80 °C until processing. Both the whole larvae and midgut bioassays were replicated independently three times as full biological replications.

### RNA extraction, library construction and sequencing

RNA was extracted using Trizol and purified by Direct-zol RNA Mini Prep kit (Zymo Research, Irving, CA). DNase treatment (ThermoFisher, Waltham, MA) was incorporated to remove genomic DNA contamination. RNA samples were checked for integrity using a fragment analyzer (University of Missouri DNA Core Laboratory). Strand-specific RNA-Seq libraries were prepared using the Illumina TruSeq HT Stranded Total RNA Library Prep Kit (Illumina, San Diego, CA), following the manufacturer’s instructions. For whole larvae and midgut respectively, 24 libraries (2 insect colonies × 2 corn lines × 2 times × 3 biological replications) were normalized, pooled and sequenced on two lanes of Illumina HiSeq2000 sequencer using 100-nucleotide pair-end protocol (Global Biologics LLC, Columbia, MO, USA).

### *De novo* assembly of transcriptome

Adapters and low quality reads were trimmed using FastqMcf (version 1.04.803, https://github.com/ExpressionAnalysis/ea-utils/blob/wiki/FastqMcf.md) and Trimmomatic (version 0.36)^49^, respectively. To remove the mtDNA, the reads were aligned to the WCR mitochondrial genome^50^ using Bowtie 2 (version 4.7.7)^51^ with default settings. Paired reads concordantly aligned with no mismatch were considered as mitochondrial reads and discarded. These steps resulted in “clean reads” for assembly and differential expression analysis.

Trinity (version r2013-11-10)^52^ was used to *de novo* assemble larval and midgut transcriptomes using cleaned reads pooled from libraries of each sample type with default setting. The unigene set of each transcriptome was obtained by removing sequence with over 95% of similarity in Blast2GO^53^ (v4.1.9). BUSCO analyses (version v3 with Insecta odb9 dataset)^37^ were performed on transcriptome assemblies and unigene sets to evaluate their quality and completeness.

### Differential expression analysis

Cleaned reads were aligned to the corresponding transcriptome unigene set using Bowtie 2 with default pair-end settings^51^. The output SAM files were converted to BAM format using SAMtools (version 0.1.20)^54^. The differential expression analysis was conducted in R (version 3.4.1)^55^. The read counts were called in R using the GenomicAlignment (version 1.6.3) and GenomicRanges (1.22.4) packages^56^. We counted only concordant alignment pairs while accepting multiple mapping reads due to the potential existence of isoforms in the unigene sets. The low expression unigenes were removed by applying filters with at least 2 count per million (CPM) over 3 samples. The differentially expressed contigs were assessed using the edgeR-robust algorithm of the edgeR package (version 3.12.1)^38,57^ with the trimmed mean of M-values (TMM) normalization method^58,59^. False discovery rate (FDR) was controlled at 0.05 by the edgeR package and was used to determine the significance of differentially expressed genes (DEGs).

### Annotation and pathway analysis

Blast2GO was used for gene annotation, enrichment and pathway analysis. Transcriptomes were annotated by BLASTX against NCBI non-redundant (NR) database using an E-value cutoff of 1.0E-3. InterProScan was used to identify protein domains from 11 member databases^60^. Gene ontology terms (GO) were assigned based on the results of BLASTX and InterProScan. Gene set enrichment analysis (GSEA) was used to determine enriched GO terms by comparing the gene list versus the entire transcriptome. The enzyme codes were assigned to predicted enzymes function based on their GO terms. Those enzymes were mapped to Kyoto Encyclopedia of Gene and Genomes (KEGG) database for pathway analysis.

## Supplementary information

Supplementary Dataset 1

Supplementary Dataset 2
